# T Cell-Derived IL-10 Determines Leishmaniasis Disease Outcome and Is Suppressed by a Dendritic Cell Based Vaccine

**DOI:** 10.1371/journal.ppat.1003476

**Published:** 2013-06-27

**Authors:** Tobias Schwarz, Katharina A. Remer, Wiebke Nahrendorf, Anita Masic, Lisa Siewe, Werner Müller, Axel Roers, Heidrun Moll

**Affiliations:** 1 Institute for Molecular Infection Biology, University of Würzburg, Würzburg, Germany; 2 Department of Dermatology, University of Cologne, Cologne, Germany; 3 Department of Experimental Immunology, The Helmholtz Centre for Infection Research, Braunschweig, Germany; 4 Bill Ford Chair of Cellular Immunology, Faculty of Life Sciences, University of Manchester, Manchester, United Kingdom; 5 Institute for Immunology, University of Dresden, Dresden, Germany; Imperial College London, United Kingdom

## Abstract

In the murine model of *Leishmania major* infection, resistance or susceptibility to the parasite has been associated with the development of a Th1 or Th2 type of immune response. Recently, however, the immunosuppressive effects of IL-10 have been ascribed a crucial role in the development of the different clinical correlates of *Leishmania* infection in humans. Since T cells and professional APC are important cellular sources of IL-10, we compared leishmaniasis disease progression in T cell-specific, macrophage/neutrophil-specific and complete IL-10-deficient C57BL/6 as well as T cell-specific and complete IL-10-deficient BALB/c mice. As early as two weeks after infection of these mice with *L. major*, T cell-specific and complete IL-10-deficient animals showed significantly increased lesion development accompanied by a markedly elevated secretion of IFN-γ or IFN-γ and IL-4 in the lymph nodes draining the lesions of the C57BL/6 or BALB/c mutants, respectively. In contrast, macrophage/neutrophil-specific IL-10-deficient C57BL/6 mice did not show any altered phenotype. During the further course of disease, the T cell-specific as well as the complete IL-10-deficient BALB/c mice were able to control the infection. Furthermore, a dendritic cell-based vaccination against leishmaniasis efficiently suppresses the early secretion of IL-10, thus contributing to the control of parasite spread. Taken together, IL-10 secretion by T cells has an influence on immune activation early after infection and is sufficient to render BALB/c mice susceptible to an uncontrolled *Leishmania major* infection.

## Author Summary

The clinical symptoms caused by infections with *Leishmania* parasites range from self-healing cutaneous to uncontrolled visceral disease and depend not only on the parasite species but also on the type of the host's immune response. It is estimated that 350 million people worldwide are at risk, with a global incidence of 1–1.5 million cases of cutaneous and 500,000 cases of visceral leishmaniasis. Murine leishmaniasis is the best-characterized model to elucidate the mechanisms underlying resistance or susceptibility to *Leishmania major* parasites *in vivo*. Using T cell-specific and macrophage-specific mutant mice, we demonstrate that abrogating the secretion of the immunosuppressive cytokine IL-10 by T cells is sufficient to render otherwise susceptible mice resistant to an infection with the pathogen. The healing phenotype is accompanied by an elevated specific inflammatory immune response very early after infection. We further show that dendritic cell-based vaccination against leishmaniasis suppresses the early secretion of IL-10 following challenge infection. Thus, our study unravels a molecular mechanism critical for host immune defense, aiding in the development of an effective vaccine against leishmaniasis.

## Introduction

Infection with intracellular protozoan parasites of the genus *Leishmania* leads to a broad range of disease manifestations in humans, ranging from an asymptomatic carrier status or localized, self-healing cutaneous leishmaniasis to disseminating visceral disease (kala azar) [1]. The outcome of infection depends on the parasite species, but is also influenced by the host immune response [2], [3].

In naturally resistant mouse strains such as C57BL/6 or C3H, IL-12, secreted mainly by dendritic cells (DC), has the essential role of inducing a Th1 immune response. The Th1 effector cytokine IFN-γ leads to an activation of infected macrophages and parasite killing. Conversely, the susceptibility of BALB/c mice has been attributed to a Th2 immune response characterized by the secretion of IL-4, IL-5 and IL-13. Accordingly, IL-4^−/−^ BALB/c mice are able to control infection with some *Leishmania major* strains at least partially [4] and BALB/c mice treated with anti-IL-4 Ab at the time of challenge exhibit a healing phenotype [5]. There is also convincing evidence that the early IL-4 response is confined largely to an oligoclonal population of CD4^+^ T cells with a Vβ4Vα8 T-cell receptor that recognize the *Leishmania* antigen LACK (Leishmania homologue of receptors for activated C kinase) [6]. However, this classical Th1/Th2 paradigm has been challenged by recent findings in humans and some mouse models: for instance, IL-4^−/−^ and IL-4Rα^−/−^ BALB/c mice are not resistant against all *L. major* strains [7], and, whereas IL-4^−/−^ and IL-4Rα^−/−^ BALB/c mice are resistant to infection with *L. amazonensis*, IL-4^−/−^ C57BL/6 and IL-4^−/−^ C3H mice are not [8], [9]. In addition, IL-4^−/−^ and IL-4Rα^−/−^ BALB/c mice are more susceptible to infection with *L. donovani* [10] and also experimental visceral leishmaniasis in C57BL/10 mice is independent of IL-4 and the associated Th2 immune response [8], [11].

In contrast, recent studies have emphasized the role of IL-10 as an important regulatory cytokine involved in parasite control in mice and humans. Originally identified as a Th2 cell-derived factor, it is now known to be secreted also by regulatory T cells (Treg), Th1 cells, CD8^+^ T cells, B cells, macrophages, DC, mast cells, eosinophils, NK cells and some cell types not belonging to the immune system [12], [13], [14]. IL-10 has broad anti-inflammatory effects on several cell types: it inhibits phagocytosis, the expression of MHC class II and co-stimulatory molecules and the secretion of proinflammatory cytokines by macrophages and DC. It also limits the ability of macrophages to kill intracellular organisms. In addition to its influence on cells of the innate immune system, IL-10 has direct suppressive effects on T cell responses [12]. Accordingly, IL-10^−/−^ mice develop excessive Th1 cell responses, resulting for example in spontaneous inflammatory bowel disease [15] or overwhelming immunopathology upon infection with parasites like *Toxoplasma gondii* [16], *Trypanosoma* [17], *Schistosoma* [18] or *Trichinella spiralis* [19].

IL-10 has also been shown to facilitate the spread of *Leishmania* parasites. IL-10^−/−^ mice on a BALB/c background were able to control infection with *L. major* [20], and IL-10^−/−^ mice on a C57BL/6 background, in contrast to their wild-type littermates, achieved sterile immunity to this parasite [21]. In humans, cutaneous leishmaniasis, visceral leishmaniasis and post-kala-azar dermal leishmaniasis have been associated with increased levels of IL-10 [22], [23], [24], [25], [26]. Thus, IL-10 undoubtedly plays a crucial role in *Leishmania* disease progression. However, a variety of cell types is able to secrete IL-10 and there is no consensus about the cellular sources contributing to the IL-10-mediated suppression of the anti-leishmanial immune response. Belkaid et al. demonstrated that parasite persistence and the maintenance of immunity to re-infection in C57BL/10 mice are dependent on the CD4^+^ CD25^+^ FoxP3^+^ Treg cell-derived IL-10 [27], [28]. In contrast, following infection of C57BL/6 mice with the *L. major* strain NIH/Sd, which produces nonhealing dermal lesions in a Th1-polarized setting, it was shown that IL-10-producing CD4^+^ CD25^−^ FoxP3^−^ Th1 cells rather than Treg cells are the major contributors to immune suppression [29]. This was also true for BALB/c IL-4 receptor-deficient mice infected with *L. major*. Similar results were obtained in a mouse model of visceral leishmaniasis, following infection with *L. donovani* [30], as well as in human visceral leishmaniasis [26]. In a BALB/c IL-10^−/−^ mouse model, however, the observed resistance to infection was attributed to the absence of IL-10 secreted by macrophages [20]. Furthermore, the overexpression of IL-10 under control of the MHC class II Ea promoter resulted in the increased susceptibility of the mice to infection with *L. major*, supporting a role for IL-10 derived from professional APC [31]. Recently, IL-10 secreting B cells have also been shown to influence the immune response in BALB/c mice infected with *L. major* [32]. Taken together, IL-10 secretion by CD4^+^ CD25^+^ FoxP3^+^ Treg cells, CD4^+^ CD25^−^ FoxP3^−^ effector T cells, B cells and professional APC has been connected with the suppression of the anti-leishmanial immune response.

In the present study, we directly investigated the contribution of IL-10 from different cellular sources to *L. major* disease progression by using mice with a selective deficiency for IL-10 in T cells [33] or macrophages and neutrophils [34], and comparing them with complete IL-10-deficient animals. The results show that the enhanced protection of complete IL-10-deficient mice is entirely attributable to the lack of T cell-derived IL-10, while macrophage- or neutrophil-derived IL-10 has no effect on disease progression. In addition, we analyzed the mechanism underlying this enhanced protection and demonstrated that the suppression of the early antigen-dependent IL-10 secretion seems to contribute to the protection mediated by DC-based vaccination against leishmaniasis [35], [36].

## Results

### T cell-specific IL-10-deficient C57BL/6 mice develop enhanced inflammation despite unaltered parasite loads early after infection with *L. major*

Although IL-10 has been shown to play a role in protective immunity against *L. major*, the relative contributions of the different cellular sources of IL-10 are not clear. To directly investigate the role of T cell-derived and macrophage-derived IL-10, IL-10^fl/fl^ CD4-Cre^+^ T cell-specific IL-10-deficient mice were compared to IL-10^fl/fl^ LysM-Cre^+^ macrophage/neutrophil-specific IL-10-deficient mice, IL-10^fl/fl^ EIIa-Cre^+^ complete IL-10-deficient mice, and IL-10^fl/fl^ Cre^−^ control mice on a C57BL/6 background. The generation of these mice and the high specificity and efficiency of the cell type-specific deletion of the first *IL10* exon have been described previously [33], [34].

**Figure 1:**
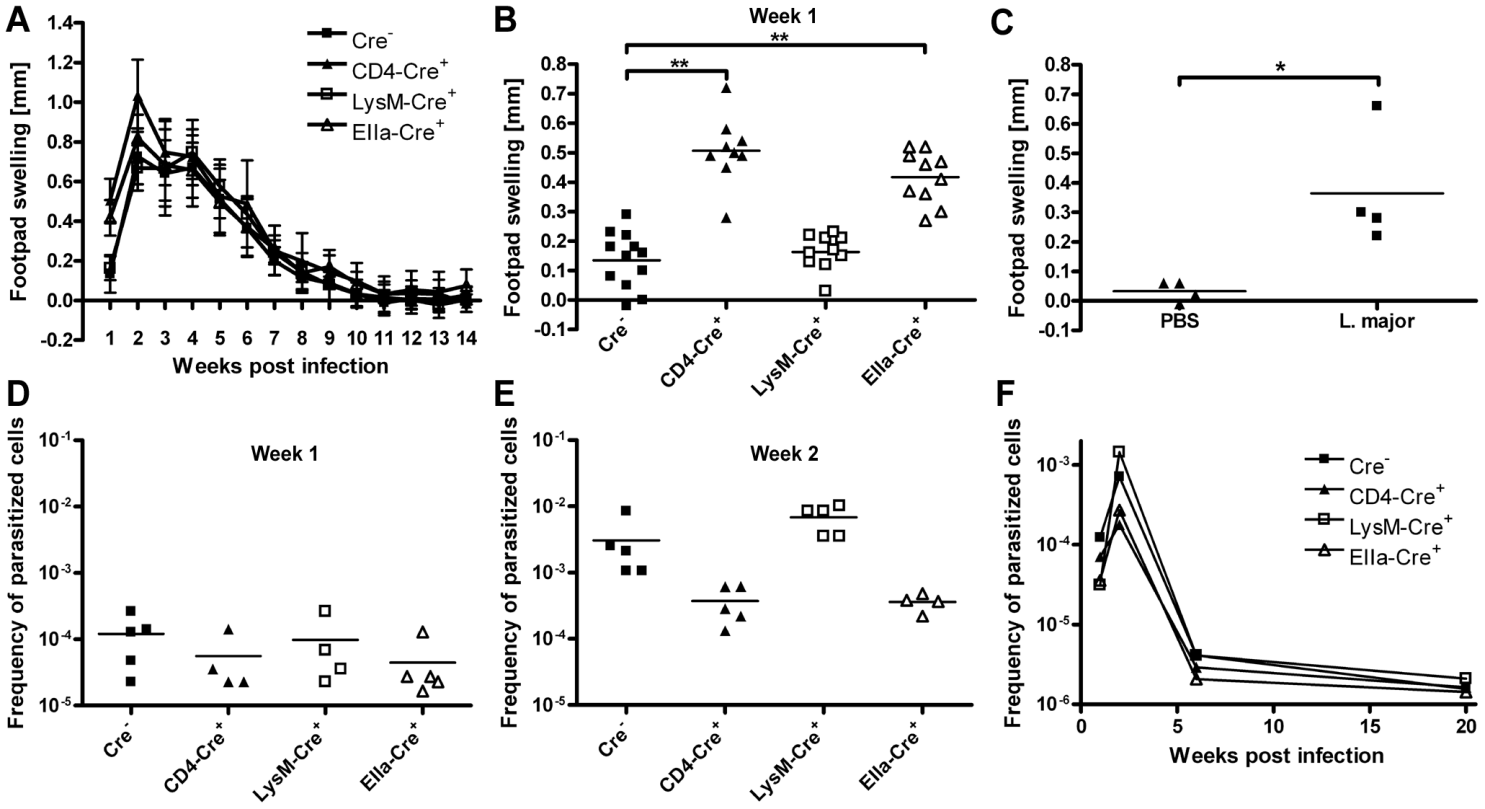
Course of infection of T cell-specific, macrophage specific and complete IL-10-deficient C57BL/6 mice. T cell-specific and complete IL-10-deficient C57BL/6 mice develop enhanced inflammation despite unaltered parasite loads 7 days after infection with *L. major*. T cell-specific (CD4-Cre^+^), macrophage/neutrophil-specific (LysM-Cre^+^), complete (EIIa-Cre^+^) IL-10-deficient and IL-10-competent control mice (Cre^−^) on a C57BL/6 background were infected with *L. major* promastigotes into the right hind footpad. The increase in size of the infected compared with the non-infected footpad was measured (**A, B and C**). Mean ± SD of 8–10 mice/group (**A**). Footpad swelling 7 days after infection (**B**). Footpad swelling of T cell-specific IL-10-deficient C57BL/6 mice 7 days after injection of PBS or *L. major* parasites (**C**). Frequency of parasitized cells in the lymph nodes draining the lesions (**D, E and F**). Each symbol represents one individual mouse (**B, C, D and E**). One representative of two independent experiments is shown (**A, B, D and E**). Each time point represents the mean of two to four independent experiments with 4–6 mice in each group and experiment (**F**). * *p*<0.05, ** *p*<0.01. Differences in parasite load did not reach statistical significance (**D, E and F**).

**Figure 2:**
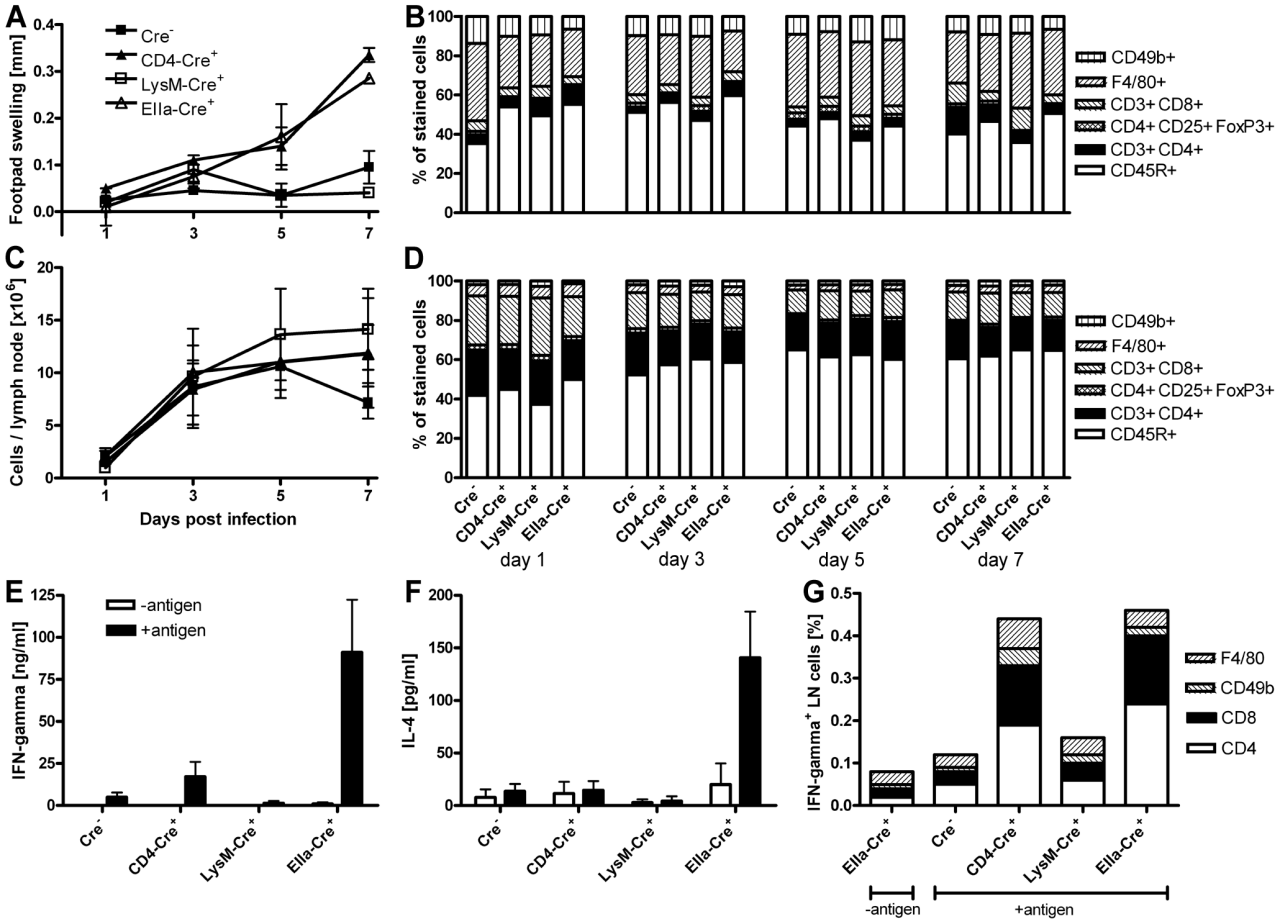
Characterization of the early inflammation in *L. major*-infected T cell-specific IL-10-deficient C57BL/6 mice. The early enhanced inflammation in T cell-specific and complete IL-10-deficient C57BL/6 mice is not associated with changes in the cell numbers but correlates with elevated secretion of IFN-γ by CD4^+^ and CD8^+^ cells. C57BL/6 IL-10 mutant mice were infected with *L. major* promastigotes into the right hind footpad. Increase in size of the infected compared with the non-infected footpads (**A**). Frequencies of cell populations in the infected feet (**B**). Cell numbers in the draining lymph nodes (**C**) and frequencies of cell populations in the draining lymph nodes (**D**). The results show the mean of two independent experiments with 2 mice per group and time point (**A to D**). Secretion of IFN-γ (**E**) and IL-4 (**F**) by draining lymph node cells 7 days after infection with *L. major*. Data show the mean ± SEM of four independent experiments with 2 mice per group and experiment (**E and F**). Draining lymph node cells of infected mice were stained for IFN-γ and the indicated surface markers; the fraction of double positive cells of all lymph node cells is shown. The results are representative of two experiments (**G**).

To investigate disease progression, these T cell-specific, macrophage/neutrophil-specific and complete IL-10-deficient mice were infected with *L. major* promastigotes into the right hind footpad and footpad swelling was monitored weekly (**Figure 1A**). Surprisingly, T cell-specific and complete IL-10-deficient mice displayed a significantly (p<0,01) increased footpad swelling, compared to macrophage/neutrophil-specific IL-10-deficient mice and Cre^−^ control animals, as soon as one week after infection (**Figure 1B**). In contrast, we could not observe any difference in footpad swelling at all later time points, including the peak of disease manifestation at 2 to 3 weeks after infection. Furthermore, there was no difference in the number of regional lymph node cells, draining the site of infection at any time point (**Figure 2C** and data not shown). To rule out that the observed early footpad swelling of the T cell-specific IL-10 deficient mice is an unspecific reaction to injection trauma, we compared footpad swelling following injection of live *L. major* promastigotes or PBS respectively. One week after injection of PBS no significant footpad swelling could be observed (**Figure 1C**). As footpad swelling reflects not only parasite replication but also local inflammation, we determined the parasite load in the regional lymph nodes of these mice at different time points after infection. Unexpectedly, we found only minor differences in the parasite load at one week after infection (**Figure 1D**), with slightly reduced parasite numbers in the T cell-specific and the complete IL-10-deficient mice, indicating that the augmented footpad swelling of these mouse strains is due to an enhanced inflammation at this early time point. However, two weeks after infection, despite comparable footpad swelling, the T cell-specific and the complete IL-10-deficient mice displayed markedly reduced parasite loads within the draining lymph nodes, compared to the macrophage/neutrophil-specific IL-10 mutant and the Cre^−^ control mice (**Figure 1E**). At later time points, parasite loads were equally low in all investigated mouse strains, consistent with the healing phenotype of the naturally resistant C57BL/6 background (**Figure 1F**).

### The early enhanced inflammation is due to an elevated secretion of IFN-γ by CD4^+^ and CD8^+^ T cells

As the influence of IL-10 on the chronic phase of *L. major* infection and especially its role for sterile immunity has already been investigated before [21], [27], [37], we further concentrated on the effects of T cell-derived IL-10 on the activation of the immune system early after infection. While all our mouse strains displayed an elevated total cell number in the draining lymph nodes already 3 days post infection, the increased footpad swelling of the T cell-specific and the complete IL-10-deficient mice was not detectable until 5 days post infection (**Figure 2A and C**). To further characterize the early enhanced inflammation of the T cell-specific IL-10 mutant mice, the relative amounts of CD4^+^ and CD8^+^ T cells, CD4^+^ CD25^+^ FoxP3^+^ Treg cells, CD45R^+^ B cells, CD49b^+^ NK cells and F4/80^+^ macrophages in the popliteal lymph nodes of infected mice and in the infected feet were analyzed by flow cytometry. The early increase in the total lymph node cell count was associated with a relative increase of B cells and a decrease of CD4^+^ T cells, CD4^+^ CD25^+^ FoxP3^+^ Treg cells, CD8^+^ T cells and macrophages (**Figure 2D**). In accordance with the uniform increase in absolute lymph node cell numbers, this shift in cell populations did not differ between the IL-10 mutant strains. All cell populations could also be found in low absolute numbers in the infected feet. However, cell type composition did not significantly differ between the IL-10 mutant strains nor change during the first week of infection (**Figure 2B**).

To investigate a functional correlate of the early increased footpad swelling of the T cell-specific and the complete IL-10-deficient mice, we restimulated lymph node cells of the different IL-10-deficient mouse strains with *L. major* lysate 7 days after infection and assayed for the production of IFN-γ, IL-4, IL-10 and IL-17. The increased footpad swelling was associated with a significantly enhanced antigen-dependent secretion of IFN-γ, while we found only a slight increase in IL-4 secretion by the complete IL-10-deficient mice (**Figure 2E and F**). No significant IL-10 or IL-17 production was detectable in any mouse strain at that time point (data not shown).

As a variety of cell types is known to be able to secrete IFN-γ, we next determined the cell population which is suppressed by the early T cell-derived IL-10. To this end, we performed intracellular cytokine staining for IFN-γ of lymph node cells of the different IL-10 mutant mouse strains 7 days after infection with *L. major* (**Figure 2G**). Surprisingly, the early enhanced secretion of IFN-γ by lymph node cells of the T cell-specific and the complete IL-10 mutant mice is not only due to antigen-specific CD4^+^ T cells, reflecting a Th1 immune response, but also to CD8^+^ T cells, whereas there was no enhanced secretion of IFN-γ by macrophages or NK cells.

### T cell-specific IL-10-deficient as well as complete IL-10-deficient BALB/c mice exhibit a healing phenotype

**Figure 3:**
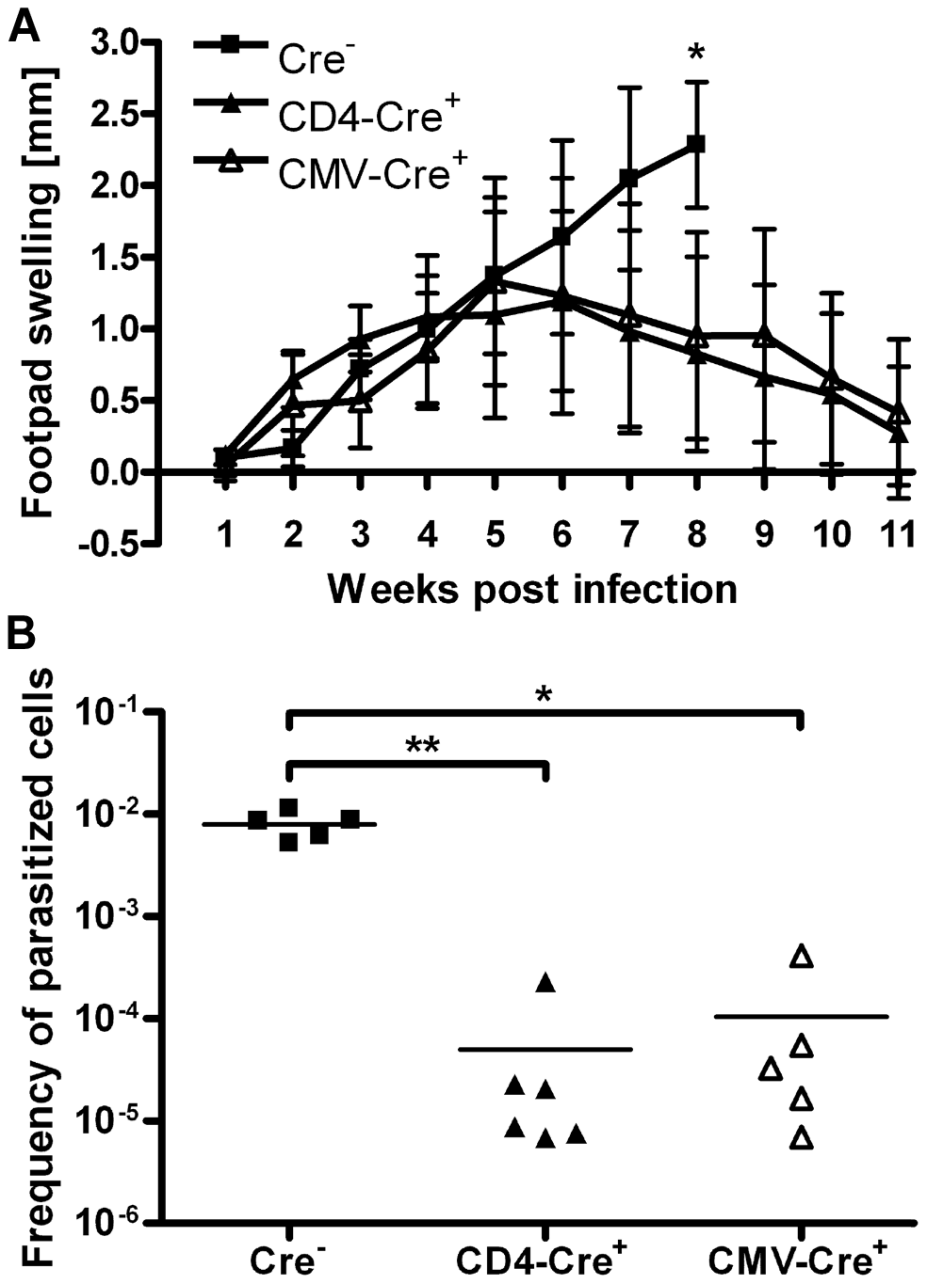
T cell-specific IL-10-deficient as well as complete IL-10-deficient BALB/c mice exhibit a healing phenotype. T cell-specific (CD4-Cre^+^) and complete (CMV-Cre^+^) IL-10 mutant BALB/c mice as well as IL-10-competent control mice (Cre^−^) were infected with *L. major* promastigotes into the right hind footpad. Lesion development (**A**); data represent the mean ± SD of 4–6 mice per group. Parasite burden in the draining lymph nodes was determined 8 weeks (Cre^−^ mice) or 11 weeks (CD4-Cre^+^ and CMV-Cre^+^ mice) post infection (**B**); each symbol represents one individual mouse. * *p*<0.05, ** *p*<0.01. The results are representative of three independent experiments.

It has been shown previously that complete IL-10-deficient mice on the naturally susceptible BALB/c background are able to control an infection with *L. major*. This has been attributed mainly to the lack of IL-10 secretion by macrophages [20]. In our model, using mice on the naturally resistant C57BL/6 background, only T cell-specific but not macrophage/neutrophil-specific IL-10-deficient mice displayed an altered disease progression. Therefore, we addressed the question whether abrogating the T cell-specific IL-10 secretion also has an effect on disease progression in the naturally susceptible BALB/c background. For this purpose, we backcrossed the C57BL/6 IL-10^fl/fl^ CD4-Cre^+^ mice onto the BALB/c background. To obtain complete IL-10-deficient mice on a BALB/c background, IL-10^fl/fl^ Cre^−^ mice were crossed with CMV-Cre^+^ mice [38]. Following infection with *L. major*, the T cell-specific IL-10 mutant BALB/c mice displayed a healing phenotype, indistinguishable from the complete IL-10-deficient BALB/c mice (**Figure 3A**). The reduced footpad swelling was accompanied by a marked control of parasite replication, as demonstrated by an up to three log reduced parasite load in the popliteal lymph nodes (**Figure 3B**).

### The early inflammation in T cell-specific IL-10-deficient BALB/c mice is associated with a mixed Th1/Th2 immune response

**Figure 4:**
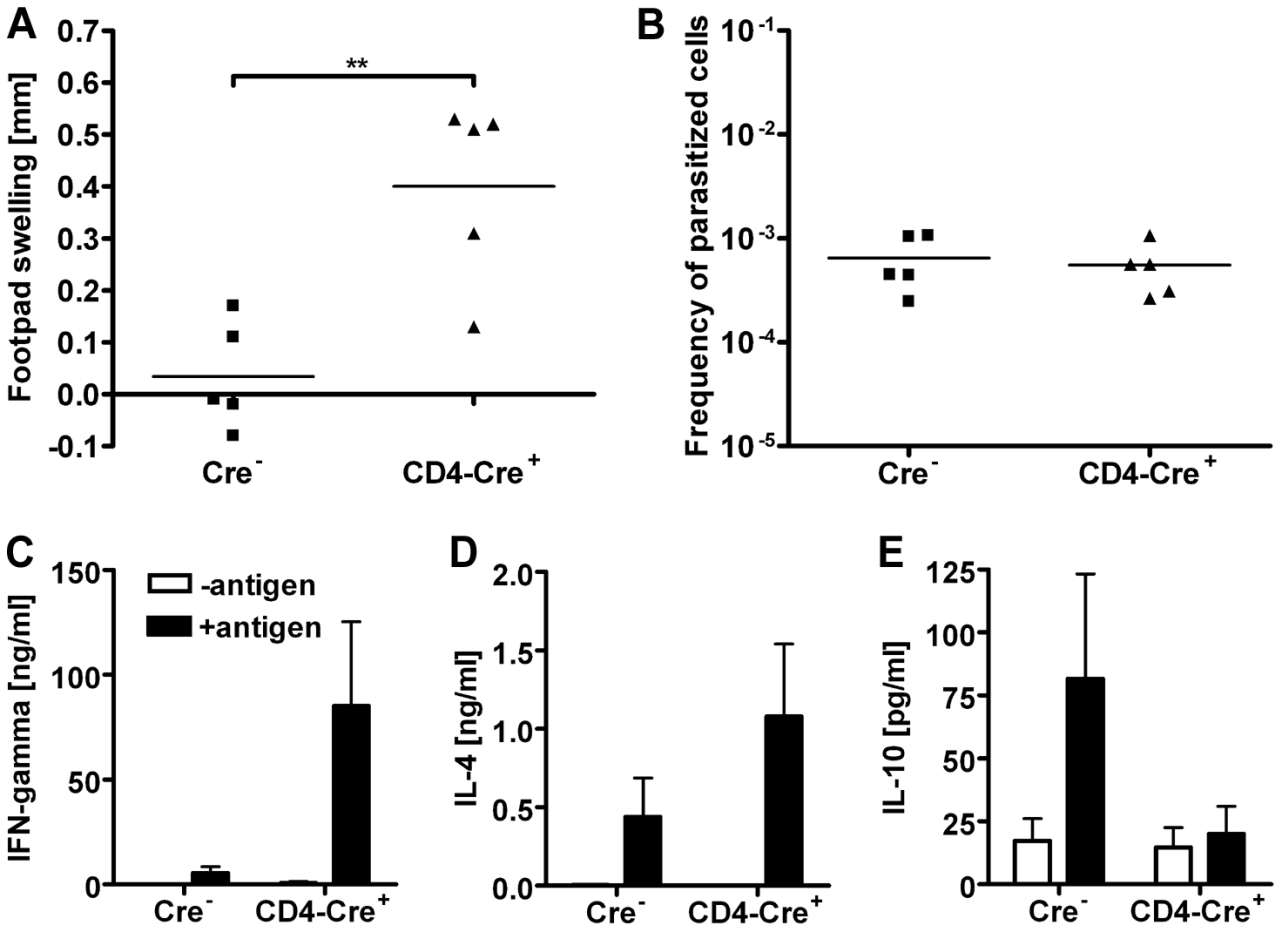
Characterization of the early inflammation in *L. major*-infected T cell-specific IL-10-deficient BALB/c mice. Enhanced inflammation in T cell-specific IL-10-deficient BALB/c mice 2 weeks after infection is associated with a mixed Th1/Th2 immune response. T cell-specific IL-10 mutant and IL-10-competent BALB/c mice were infected with *L. major* promastigotes into the right hind footpad. Increase in size of the infected footpad 2 weeks after infection (**A**). Frequency of *L. major*-infected cells in the draining lymph nodes (**B**). Cytokine secretion upon restimulation of lymph node cells with *Leishmania* antigen *in vitro* (**C, D and E**). Each symbol represents an individual mouse (**A and B**); the results are representative of three independent experiments. Data show the mean ± SEM of three independent experiments with 5–6 mice per group and experiment (**C, D and E**). ** *p*<0.01.

Interestingly, the T cell-specific as well as the complete IL-10-deficient BALB/c mice also showed an increased footpad swelling early after infection, comparable to the respective IL-10 mutant mice on a C57BL/6 background (**Figure 3A** **and** **4A**). As the complete IL-10-deficient BALB/c mice were breeding very poorly, and no differences could be detected between the T cell-specific and the complete IL-10-deficient mice, only T cell-specific IL-10-deficient and Cre^−^ control mice were used for the further experiments. Further investigation of the parasite load and immune response of BALB/c mice two weeks after infection with *L. major* showed that despite an increased footpad swelling of the T cell-specific IL-10-deficient mice (**Figure 4A**) there were again no differences in parasite load at that early time point (**Figure 4B**). To determine the type of immune response underlying the increase in early footpad swelling in mice of the BALB/c background, cytokines in the supernatants of cultured lymph node cells were determined by ELISA. Surprisingly, not only the Th1 marker cytokine IFN-γ, but also the Th2 marker cytokine IL-4 was significantly upregulated in the T cell-specific IL-10 mutant mice (**Figure 4C–E**).

### The T cell-derived IL-10 mediating suppression of the early inflammatory response is mainly secreted by FoxP3^−^/CD25^−^ cells in the draining lymph nodes and FoxP3^+^/CD25^+^ cells in the infected footpads

**Figure 5:**
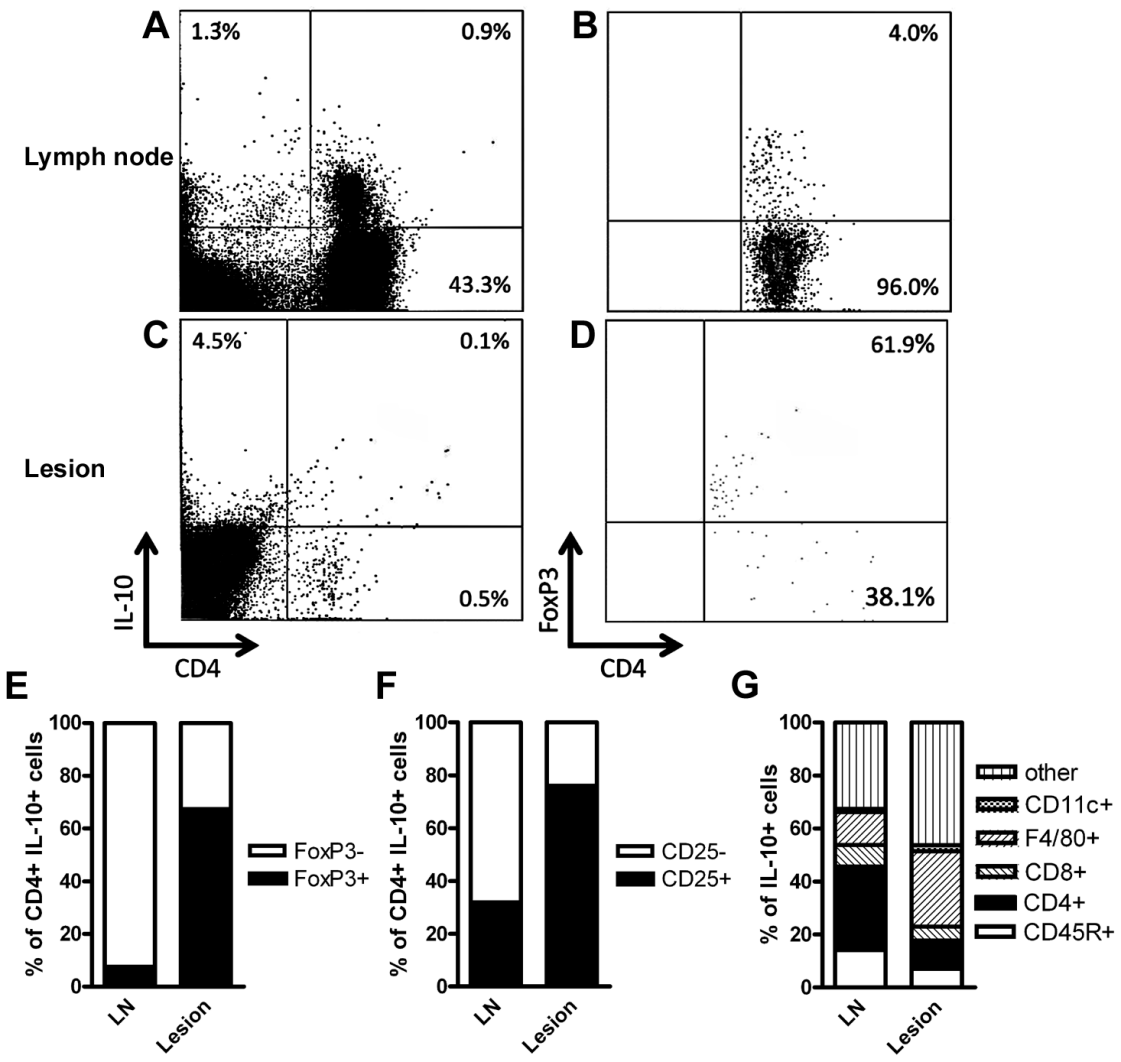
Populations of IL-10 secreting leukocytes in lesions and draining lymph nodes early after infection with *L. major*. Following infection with *L. major*, the early secretion of IL-10 by CD4^+^ T cells in the draining lymph nodes is primarily due to FoxP3^−^/CD25^−^ T cells. Within the infected footpads, however, the low absolute number of IL-10 secreting CD4^+^ T cells is predominantly FoxP3^+^/CD25^+^. The remaining IL-10 secreting leukocytes within the infected footpads are primarily macrophages, whereas in the draining lymph nodes, also B cells and CD8^+^ T cells contribute to the overall IL-10 secretion Wild-type BALB/c mice were infected with *L. major* promastigotes into the hind footpads and 2 weeks later, cells from draining lymph nodes and lesion-derived cells were prepared. IL-10-secreting cells were identified using a cytokine secretion assay (**A and C**), and the proportion of CD4^+^ FoxP3^+^ (**B, D and E**), CD4^+^ CD25^+^ (**F**), CD8^+^, CD45^+^, F4/80^+^ or CD11c^+^ cells (**G**) was determined. The means of 4 (**E**) or 3 (**F and G**) independent experiments with 3 mice each are given.

Since IL-10 secretion by CD4^+^ CD25^+^ FoxP3^+^ Treg cells [27], [28], [39] as well as CD4^+^ CD25^−^ FoxP3^−^ Th1 cells [29], [30], [40] has been implicated in the control of chronic infection with *Leishmania*, it was important to determine the T cell population secreting IL-10 early after infection. To this end, naïve wild-type BALB/c mice were infected with *L. major* promastigotes in the hind footpad. Two weeks later, the IL-10-secreting cells within the draining popliteal lymph nodes and the footpad lesions were identified by a cytokine secretion assay. Staining for CD4 and FoxP3 or CD25 showed that the majority of CD4^+^ IL-10-secreting cells in the draining lymph nodes were FoxP3^−^ and CD25^−^. In the infected footpads, there was only a small number of CD4^+^ IL-10-secreting cells, the majority of which were FoxP3^+^ and CD25^+^ (**Figure 5A–F**).

Furthermore, T cells represented only a small proportion of the IL-10 secreting cells. Staining for CD8, CD45R, CD11c and F4/80 revealed that all these cell populations contribute to IL-10 secretion in the draining lymph nodes and the footpads of *L. major*-infected wild-type BALB/c mice, emphasizing the profound differences in the effects of IL-10 from different cellular sources (**Figure 5G**).

### DC-based vaccination decreases the early secretion of IL-10 following infection with *L. major*

**Figure 6:**
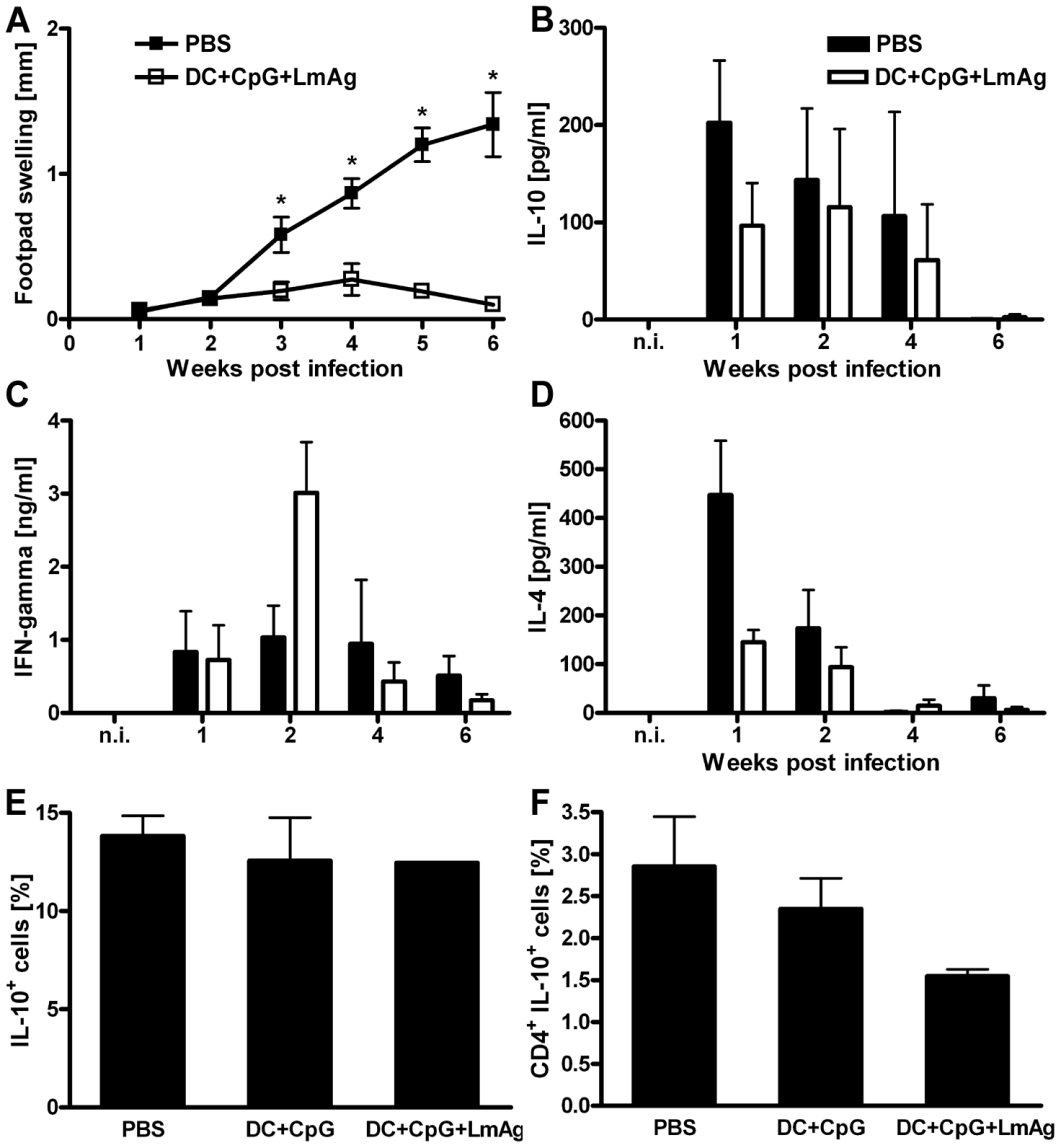
DC-based vaccination decreases the early secretion of IL-10 by CD4^+^ T cells following infection with *L. major* Wild-type BALB/c mice were immunized *in vivo* with fragmented BMDC that had been treated *in vitro* with *Leishmania* antigen and CpG oligodeoxynucleotides and were challenged with *L. major* promastigotes 1 week later. Control mice were mock-treated with PBS (**A to D**) or CpG activated BMDC (**E and F**). Increase in size of the infected compared with the non-infected footpad (**A**). Cytokine secretion of the draining lymph node cells upon restimulation with *Leishmania* antigen, measured by ELISA (**B, C and D**). Number of IL-10-secreting cells of all lymph node cells (**E**) and number of CD4^+^ IL-10-secreting cells of all lymph node cells (**F**) one week after infection. Data represent the mean ± SEM of three independent experiments with 2 mice per group and time point (**A to D**) or two independent experiments with 5 mice per group (**E and F**). * p<0.05.

According to current paradigm, a maximal secretion of the proinflammatory cytokines IFN-γ, TNF and IL-2 by antigen-specific T cells after pathogen contact is the major prerequisite for successful vaccination against *L. major* [41]. The early increase in inflammation accompanied by a better control of parasite replication in both T cell-specific IL-10-deficient C57BL/6 and BALB/c mice following infection with *L. major* raised the question whether a reduced antigen-specific secretion of IL-10 may contribute to effective vaccination against the disease. To test this hypothesis, we used a DC-based vaccination protocol which has been shown to induce highly effective and solid immunity against *L. major* infection [35], [36], [42]. To explore the effect of DC-mediated protection on the early secretion of IL-10 by antigen-specific T cells, we compared the cytokine activity of cells in the lymph nodes draining the lesions of vaccinated versus non-vaccinated mice at different time points after infection with *L. major*. Interestingly, the only significant differences in the antigen-dependent cytokine secretion between vaccinated and non-vaccinated mice were indeed observed the first two weeks after infection. In the very first week after infection the vaccinated mice produced less than half the amount of IL-10 and IL-4 compared to the non-vaccinated mice (**Figure 6B and D**). At this time point, we did neither observe a difference in footpad swelling (**Figure 6A**) nor in parasite load between the two groups (data not shown). Already two weeks after infection the vaccinated mice showed a significant increase in IFN-γ secretion (**Figure 6C**).

To further investigate if the reduced IL-10 secretion by lymph node cells of vaccinated mice one week after infection is accompanied by a reduced number of IL-10-secreting CD4^+^ T cells, an IL-10 cytokine secretion assay was performed. Interestingly, despite comparable numbers of all IL-10-secreting lymph node cells between the vaccinated and the non-vaccinated mice (**Figure 6E**), the number of IL-10-secreting CD4^+^ T cells was reduced by half in the successfully vaccinated mice (**Figure 6F**).

## Discussion

The cytokine IL-10 has a major impact on the regulation of inflammation and the progression of a multitude of infectious diseases (reviewed in [14], [43], [44]). During infection with intracellular parasites of the genus *Leishmania*, IL-10 undoubtedly plays an important role in disease development and pathogen persistence [20], [21], [22], [23], [24], [27], [37], [39]. However, the cellular source of immune suppressive IL-10 is less clear, as activated effector T cells [29], [30], [40], Treg cells [27], [28], [39], macrophages [20], [31], [45], DC [42] as well as B cells [32] have been implicated in inhibiting the effective control and clearance of the parasite. Furthermore, the influence of IL-10 on the early induction of an immune response against *L. major* has been scarcely investigated. In the present study, we used T cell-specific, macrophage/neutrophil-specific and complete IL-10-deficient mice to directly address these questions. As previous studies on the role of IL-10 in leishmaniasis were mostly investigating differences in disease progression on the naturally resistant C57BL/6 background, this study is the first to show that disabling the secretion of IL-10 by T cells is sufficient to render otherwise susceptible BALB/c mice resistant to an infection with the parasite. The healing phenotype is accompanied by an elevated specific inflammatory immune response very early after infection. We further show that DC-based vaccination against leishmaniasis suppresses the early secretion of IL-10 following challenge infection.

Following infection with *L. major*, the T cell-specific and complete IL-10-deficient mice on the C57BL/6 as well as the BALB/c background are characterized by an increased early inflammation, which is due to the enhanced secretion of IFN-γ by CD4^+^ as well as CD8^+^ T cells. This finding is in accordance with recent data of low dose infection models in C57BL/6 mice, where CD8^+^ T cells via secretion of IFN-γ significantly contribute to the induction of protection against *L. major* [46], [47]. In contrast, these effects have not been observed in earlier studies with high dose infection of MHC class II^−/−^ or β2-microglobulin^−/−^ C57BL/6 mice [48].

Although a direct inhibitory effect of IL-10 on T cells has been described [12], an increased secretion of IL-12p70 by lymph node cells of the T cell-specific and complete IL-10-deficient mice early after infection (data not shown) strongly argues for an indirect activation of CD4^+^ and CD8^+^ T cells via APC [49]. Similarly, the infection of IL-10^−/−^ mice with the intracellular parasite *T. gondii* leads to enhanced IL-12 production by APC, resulting in an overwhelming and lethal Th1 immune response [16]. Interestingly, the only difference between the T cell-specific and the complete IL-10-deficient C57BL/6 mice occurred upon antigen restimulation of draining lymph node cells seven days post infection, resulting in an increased secretion of IFN-γ and IL-4 by the lymph node cells of the complete IL-10-deficient mice. As the number of IFN-γ-secreting T cells did not differ between the two mouse strains this could emphasize the importance of the T cell-derived cytokines on disease progression. We can not rule out, however, that the increased secretion of IFN-γ and IL-4 by the IL-10 deficient cells is a result of the *in vitro* incubation of the complete cell suspensions.

At very late stages of infection the differences between individual mouse strains were less prominent than in previous studies [21], [27], [29]. These differences may be explained by different substrains of *L. major* used for the experiments, as this was shown to substantially influence disease progression [37]. Furthermore, the dose of parasites inoculated, the infection site as well as the injection route (needle challenge vs. sand fly bite) are known to influence *Leishmania* disease progression and vaccine efficiency [50]. To further elucidate the role of T cell-derived IL-10 in long-term infection, the conditional IL-10-deficient mice were backcrossed on the naturally susceptible BALB/c background. Intriguingly, T cell-specific IL-10-deficient, like complete IL-10-deficient BALB/c mice [20], showed a protective phenotype. To the best of our knowledge, we showed for the first time that suppression of the T cell-specific IL-10 production not only improves the outcome of disease in naturally resistant mice, but even reverses progredient disease in susceptible mice into complete parasite control. This healing phenotype was associated with an increased inflammation early after infection, similar to T cell-specific IL-10-deficient C57BL/6 mice. On the BALB/c background, however, the inflammatory response was of a mixed Th1/Th2 type, characterized by an elevated secretion of IFN-γ and IL-4. Our findings are in accordance with previous reports that antibody-mediated depletion of CD25^+^ cells and the reconstitution of SCID mice with splenocytes depleted of CD4^+^ CD25^+^ T cells leads to an early burst of IL-4 and a slight increase in IFN-γ secretion in the draining lymph nodes of mice on a BALB/c background [51], [52]. However, this depletion led to disease exacerbation, which was attributed to the augmented early Th2 response, while the T cell-specific IL-10 mutant mice used in the present study were able to control the disease, thus indicating that the general down-regulation of the immune response by T cell-derived IL-10 is more relevant to disease outcome than the balance between a Th1 and Th2 immune response.

The macrophage/neutrophil-specific IL-10-deficient mice on a C57BL/6 background never showed any significant difference in disease progression compared to IL-10-competent control mice, neither early after infection nor at any later time point. These findings contradict the model of an autocrine IL-10-based inhibition of macrophages due to the activation of Fcγ-receptors by antigen-antibody complexes as proposed by Mosser and colleagues [20], [45]. The importance of macrophage derived IL-10, secreted following IgG-FcγR engagement, is further called into question by the finding that mice lacking IgG1 are more resistant to infection with *L. mexicana* [53]. The studies by Mosser and colleagues, however, were performed with mice on the naturally susceptible BALB/c background, whereas we could examine only macrophage/neutrophil-specific IL-10 mutant mice on the resistant C57BL/6 background. Surprisingly, at later time points after infection, macrophage/neutrophil-specific IL-10-deficient mice had slightly higher parasite loads than IL-10-competent mice, while complete IL-10-deficient mice had somewhat higher parasite loads than the T cell-specific IL-10-deficient mice on both the C57BL/6 and the BALB/c background, although these differences never reached statistical significance.

A cytokine secretion assay 7 days after infection with *L. major* revealed only a very small number of IL-10-secreting CD4^+^ cells in the draining lymph nodes. These appeared to be mainly FoxP3^−^, suggesting activated effector T cells as the primary source for the T cell-derived IL-10. These results are in accordance with the above-mentioned studies which demonstrated a disease exacerbation in BALB/c mice depleted of CD25^+^ Treg cells [51], [52]. In other mouse strains and in studies of human leishmaniasis, however, both CD4^+^CD25^−^FoxP3^−^ as well as CD4^+^CD25^+^FoxP3^+^ T cells have been implicated in the long-term control of infection and development of sterile immunity [27], [28], [29], [30], [39], [40]. To directly address this question, Treg cell-specific IL-10 mutants, which have recently been generated in our lab [54] could be used in a future study.

Apart from T cells and macrophages, a variety of other cell types is able to secrete IL-10 [12] and could be found in the feet and draining lymph nodes of *L. major* infected mice. Infection of mice with *Leishmania* parasites is known to induce secretion of IL-10 by neutrophils. Our finding that disease progression is unaltered in the macrophage/neutrophil-specific IL-10 mutant mice, however, is in accordance with data showing that early after infection with *L. major*, secretion of IL-10 by neutrophils of C57BL/6 mice is higher than by neutrophils of susceptible BALB/c mice [55]. In an experimental model of visceral leishmaniasis, also NK cells were recently shown to produce IL-10 at late stages of infection, thus reducing host resistance [56]. Furthermore, regulatory B cells have been defined, which downregulate immune responses mainly by secretion of IL-10. Notably, B cells secreting IL-10 have been shown to shape the development of Th2 immune responses and disease course in BALB/c mice infected with *L. major* [32]. Moreover, the greater susceptibility of CBA/J mice to *L. amazonensis* compared to *L. major* was found to coincide with an increased frequency of IL-10-positive splenic B cells [57]. Although these findings do not provide a clear proof, an effect of B cell-derived IL-10 on *Leishmania* infection cannot be ruled out. Furthermore, DC play a prominent role in orchestrating the induction of immune responses against pathogens and, thus, might contribute to susceptibility to infection by IL-10 secretion. Recently, Owens et al. demonstrated that during chronic *L. donovani* infection splenic CD11c^hi^ DC acquire a regulatory profile with elevated secretion of IL-27p28 and IL-10. Following therapeutic depletion of all CD11c^+^ cells, enhanced host resistance and reduced disease pathology could be observed, accompanied by a diminished development of IL-10-producing CD4^+^ T cells [58]. Therefore, it would be tempting to investigate *Leishmania* disease progression in DC-specific IL-10-deficient mice.

The finding that the early antigen-specific secretion of IL-10 by T cells influences the progression and outcome of leishmaniasis has clinically relevant implications. It clearly shows that an efficient vaccine against leishmaniasis should not only induce IFN-γ-, TNF- and IL-2-secreting memory T cells [41], but also needs to prevent the development of antigen-specific IL-10-secreting T cells. Along that line, a DC-based vaccination approach developed in our lab significantly reduced the antigen-specific production of IL-10 early after infection. Consistent with our data, the ratio of IFN-γ/IL-10 was a reliable pre-challenge indicator of vaccine success in a heterologous prime-boost vaccination approach with two different antigens that both induced low levels of IL-4 [59]. In addition, recent reports demonstrated that the protection mediated by a non-persistent parasite vaccine was accompanied by a restriction of the early IL-10 production [60], that IL-10 may account for the lack of efficiency of killed parasite vaccines [61], and that IL-10 influenced the magnitude and quality of the Th1 response following immunization with leishmanial proteins and CpG [62]. It is not possible, however, to quantitatively distinguish the importance of the early reduced IL-10 secretion from the likewise reduced IL-4 secretion and increased IFN-γ secretion following infection.

Taken together, we showed that the early secretion of IL-10 by antigen-specific FoxP3^−^ T cells suppresses the development of an inflammatory response following infection with *L*. *major*. Thus, IL-10 secretion by T cells is not only involved in the long-term control of infection and prevention of overwhelming immune activation, but also has a crucial effect on immune activation early after infection, influencing disease outcome and vaccine efficiency.

## Materials and Methods

### Ethics statement

All mice were kept under specific pathogen-free conditions. Mice experiments were performed in strict accordance with the German Animal Welfare Act 2006 (TierSchG) and the animal protocol was approved by the government of Lower Franconia (permission no. 55.2-2531.01-16/09).

### Mice

C57BL/6 IL-10^fl/fl^ CD4-Cre^+^ and IL-10^fl/fl^ LysM-Cre^+^ mice were generated as previously described [33], [34], [63]. C57BL/6 IL-10^fl/fl^ CD4-Cre^+^ mice were backcrossed onto the BALB/c background for 7 generations. C57BL/6 IL-10^fl/fl^ EIIa-Cre^+^ mice and BALB/c IL-10^fl/fl^ CMV-Cre^+^ mice were generated by crossing the respective IL-10^fl/fl^ Cre^−^ animals with C57BL/6 EIIa-Cre^+^ mice [64] or BALB/c CMV-Cre^+^ mice [38] (The Jackson Laboratory, Bar Harbor). Age- and sex-matched IL-10-deficient and IL-10^fl/fl^ Cre^−^ mice were used as controls. Female wild-type BALB/c mice were purchased from Charles River Breeding Laboratories (Sulzfeld, Germany) and were 6 to 8 weeks old at the onset of the experiments. All experiments were conducted according to the German animal protection law.

### Parasites and reagents

The cloned virulent *L. major* isolate (MHOM/IL/81/FE/BNI) was maintained by passage in BALB/c mice. Promastigotes were grown in blood agar cultures. For the preparation of parasite lysate, stationary-phase promastigotes were subjected to six cycles of rapid freezing and thawing.

### Infection of mice and analysis of the course of disease

Mice were infected intradermally with 2×10^5^ (BALB/c) or 2×10^6^ (C57BL/6) stationary-phase *L. major* promastigotes into the right hind footpad. The course of infection was monitored by measuring the increase in footpad size of the infected versus the non-infected footpad. The frequency of parasitized cells in the popliteal lymph nodes of infected mice was determined by limiting dilution analysis as described elsewhere [65]. In brief, lymph nodes draining the site of infection of individual animals were passed through a 70-µm cell strainer to obtain single cell suspensions and cells were washed in PBS. Serial 1∶4 dilutions were prepared and 4 replicates per dilution and mouse were plated in 96-well blood agar plates and incubated for 10 days at 27°C, 5% CO_2_ in a humidified atmosphere. The frequency of *L. major*-infected cells was calculated at a fraction of 37% of negative culture wells.

### Flow cytometric analysis and intracellular cytokine staining

For flow cytometry, popliteal lymph nodes draining the site of infection and infected footpads were dissected and passed through a 70-µm cell strainer. Single cell suspensions were pretreated with anti-CD16/CD32 Fc block (clone 2.4G2; BD Pharmingen, Heidelberg, Germany) and subsequently stained with fluorochrome-conjugated mAb directed against CD3 (145-2C11), CD4 (RM4-5), CD8 (53-6.7), CD49b (DX5), CD45R (RA3-6B2) (all BD Pharmingen), or biotinylated mAb directed against F4/80 (BM8; Caltag/Invitrogen, Karlsruhe, Germany), followed by PE-Cy5-labeled streptavidin conjugate (BD Pharmingen). Intracellular staining for FoxP3 (clone FJK-16s; eBioscience, San Diego) was performed according to the manufacturer's instructions. For intracellular cytokine staining, single cell suspensions of lymph nodes were cultured overnight in the absence or presence of *Leishmania* lysate (equivalent to 10 parasites/cell). Cytokine excretion was blocked by addition of 2 µM monensin (BD Pharmingen) for 4 h. Following staining of surface markers, cells were fixed with 4% paraformaldehyde in PBS, permeabilized with 0.1% saponin/1% FCS in PBS, and stained with PE-conjugated anti-mouse IFN-γ-mAb (XMG1.2; BD Pharmingen). To identify IL-10-secreting cells, a cytokine secretion assay (Miltenyi Biotec, Bergisch Gladbach, Germany) was performed according to the manufacturer's instructions. Samples were analyzed on a FACSCalibur flow cytometer (BD Pharmingen), using CellQuestPro software.

### Determination of cytokine production

Popliteal lymph nodes draining the infected footpads were collected and single-cell suspensions (1 or 2×10^6^ cells/ml as indicated) were cultured in the absence or presence of *Leishmania* lysate (equivalent to 10 parasites/cell) for 72 h. Culture supernatants were harvested for determination of the cytokines IL-2, IL-4, IL-10, IL-17 and IFN-γ by sandwich ELISA as described previously [42]. The detection limits were 8 pg/ml for IL-2, IL-4, and IL-17, 16 pg/ml for IL-10, and 100 pg/ml for IFN-γ.

### DC-based vaccination of mice

Preparation of bone marrow-derived DC (BMDC) and vaccination of mice was performed as described previously with slight modifications [36], [66]. In brief, freshly prepared bone marrow cells from 6- to 10-week-old female BALB/c mice were cultured in RPMI 1640 medium (GIBCO Invitrogen) supplemented with 10% heat-inactivated FCS, 2 mM L-glutamine, 10 mM HEPES buffer, 60 µg/ml penicillin, 20 µg/ml gentamycin and 200 U/ml GM-CSF (PeproTech, London, UK). Fresh medium containing GM-CSF was added to the cultures at days 3 and 6. After 10 days, non-adherent cells were collected and cultured at a density of 1×10^6^ cells/ml with *L. major* lysate (equivalent to 30 parasites/cell) and the CpG oligodeoxynucleotide 1668 (5′-TCCATGACGTTCCTGATGCT-3′, Qiagen Operon, Cologne, Germany) at 25 µg/ml for 16 h. Antigen-loaded BMDC were washed twice with PBS, and subjected to four cycles of rapid freezing and thawing. Mice were immunized i.v. with an equivalent of 5×10^5^ BMDC per animal. Control mice were treated with PBS only. One week after vaccination, mice were challenged intradermally with 2×10^5^ stationary-phase *L. major* promastigotes into the right hind footpad.

Cytokine secretion was measured by ELISA as described above. For ease of viewing, the values for cytokine secretion without antigen re-stimulation were subtracted from the values for the antigen-dependent cytokine secretion.

### Statistical analysis

Data were analyzed using the GraphPad Prism 4.00 software. All cytokine secretion data is shown as the mean of 3–4 independent experiments. In these cases no statistical analyses were performed. Footpad swelling following *Leishmania* infection as well as parasite frequencies are displayed per individual mouse with 4–12 mice each group. In these cases statistical analyses were performed. For the analyses of differences between more than two independent groups the Kruskal-Wallis-test followed by Dunn's posttest was performed. For comparison of two independent groups the unpaired *t*-test was used. For comparison of two groups with data from independent experiments the paired *t*-test was used. Differences were considered significant at *p*<0.05.
